# Continued Root Formation after Delayed Replantation of an Avulsed Immature Permanent Tooth

**DOI:** 10.1155/2014/832637

**Published:** 2014-05-08

**Authors:** Nima Moradi Majd, Heidar Zohrehei, Alireza Darvish, Hamed Homayouni, Mamak Adel

**Affiliations:** ^1^Department of Endodontics, Dental School, Qazvin University of Medical Sciences, Qazvin 34157-59811, Iran; ^2^Dental Research Center, Shahid Beheshti University of Medical Sciences, Evin, Tehran 1983963113, Iran

## Abstract

*Introduction.* Tooth avulsion in the young permanent dentition is a frequent finding, and its prognosis depends on the treatment of the avulsed tooth before replantation, the extra-alveolar time, the storage medium, and the patient's general health. The present report describes management of an immature avulsed lower central incisor 90 minutes after the accident. *Methods.* A right lower central incisor of a 7-year-old girl was avulsed, and it was soaked in a glass of milk. 90 minutes after avulsion, replantation was performed, and the tooth was splinted; but after two weeks the replanted tooth's pulp was necrotic. Thus, endodontic treatment was performed and root canal was filled using a calcium hydroxide and iodoform paste (Metapex). Three months later, the intracanal medication was washed out and the canal was sealed using an apical plug of calcium enriched mixture (CEM) cement. *Results.* 20 months after replantation the tooth was completely asymptomatic, with physiologic mobility. Also, continued root formation including an apical segment beyond the artificial apical plug was observed. *Conclusion.* Creation of an appropriate apical barrier following the disinfection of root canal system promoted continued root-end growth in a replanted immature permanent tooth.

## 1. Introduction

Traumatic injury to the young permanent dentition is a frequent finding [1]. Avulsion of permanent teeth usually occurs in children aged 8–10 years, due in part to short and incomplete roots and resilient periodontium and bone [2]. Although multiple avulsions and involvement of alveolar bone have been reported occasionally, single tooth avulsion is most common [1].

Prognosis depends on the treatment of the avulsed tooth before replantation, the extra-alveolar time, the storage medium, and the patient's general health [3, 4].

The following complications can occur after the teeth avulsion: pulp necrosis, damage to attachment tissue, and small localized destruction of cementum [5].

According to the guidelines which have been published by the International Association of Dental Traumatology (IADT) [6], treatment of an avulsed tooth depends upon the extra-alveolar dry time. In cases of open apices or partially developed roots with extraoral dry time less than sixty minutes, the avulsed teeth should be replanted immediately to allow revascularization [7].

Complete root development after pulp revascularization of the replanted immature permanent teeth has been reported several times [8–10], but continued root formation after root canal treatment of a replanted immature tooth has seldom been documented [11].

The present report describes management of an immature avulsed lower central incisor 90 minutes after the accident.

## 2. Case Report

A healthy 7-year-old girl was referred to the Endodontic Department of Qazvin School of Dentistry. According to her mother, the girl's right lower central incisor was avulsed ninety minutes earlier, while she was playing. The tooth had been soaked in a glass of milk.

**Figure 1:**
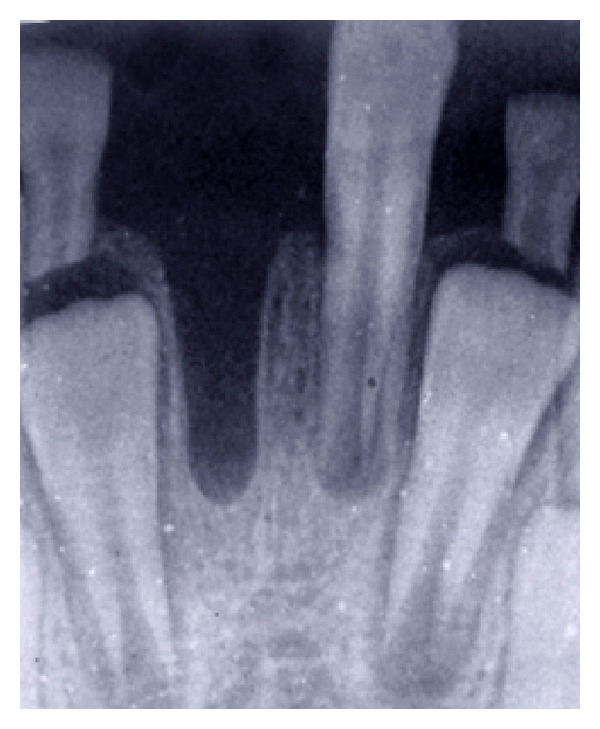
Preoperative radiograph showing the intact socket of right lower central incisor.

Intraoral examination revealed neither soft tissue laceration nor other teeth crown fracture. Periapical radiographic investigation did not show any evidence of associated hard tissue injury (Figure 1). Also, the avulsed tooth was examined, and it was found that it has intact structure with wide apical foramina.

**Figure 2:**
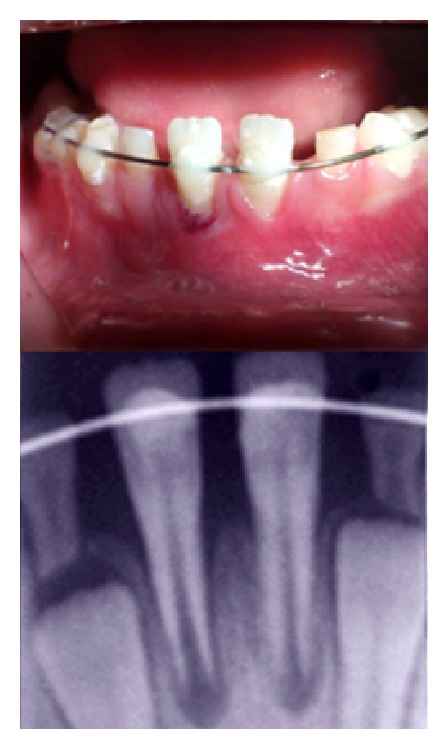
The right lower central incisor was splinted using orthodontic wire.

The avulsed tooth was soaked in doxycycline (1 mg/20 mL) (Hakim Pharmaceutical Co., Tehran, Iran) for five minutes. At the same time, the socket was carefully debrided with physiologic saline solution. After that, under local anesthesia, the tooth was gently replanted, and then it was splinted using orthodontic wire (Figure 2). The patient was prescribed a chlorhexidine gluconate mouth rinse and 3 × 250 mg amoxicillin daily for a week.

**Figure 3:**
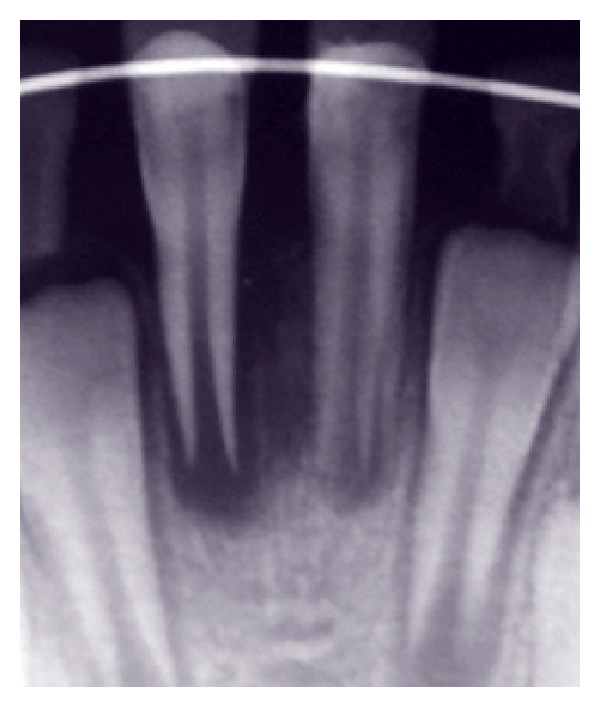
Two weeks after replantation, periapical radiolucency around the replanted tooth has increased considerably.

After two weeks, another periapical radiograph was taken and it was found that periapical radiolucency around the replanted tooth has considerably increased (Figure 3). Therefore, we decided to perform apexification. Thus, before removing splint, under local anesthesia, access cavity was prepared and canal was instrumented carefully using manual stainless steel files (Dentsply Maillefer, Switzerland) and irrigated with ten mL of sodium hypochlorite 2.5%; then, the root canal space was dried with sterile paper points (ARIADENT, Tehran, Iran) and filled using a calcium hydroxide and iodoform paste (Metapex) (META BIOMED Co. Ltd., South Korea).

In order to place the Metapex, the syringe was inserted into the canal, and paste was pressed down into the pulp space. When the paste flowed back from the root canal into the access cavity, the syringe was slowly withdrawn. Then, tooth was restored temporarily.

**Figure 4:**
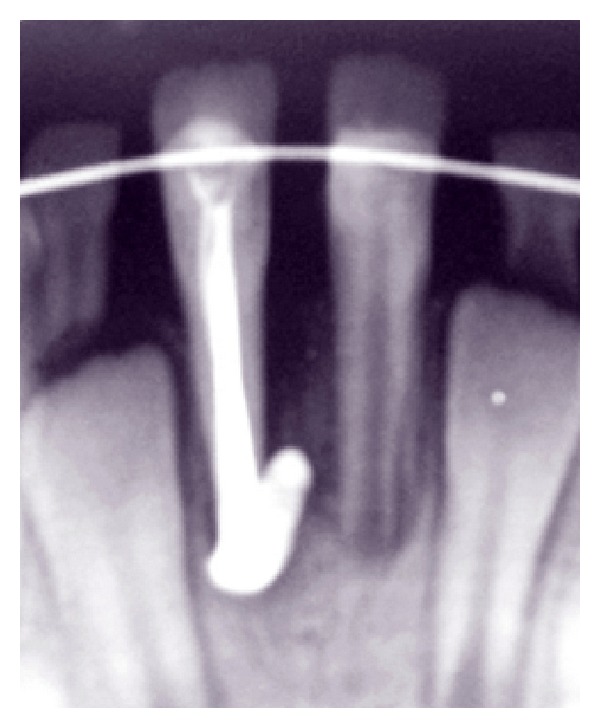
Root canal was filled using a calcium hydroxide and iodoform paste (Metapex).

After taking a periapical radiograph, it was found that, due to open apex, a part of Metapex was extruded from root canal space (Figure 4).

**Figure 5:**
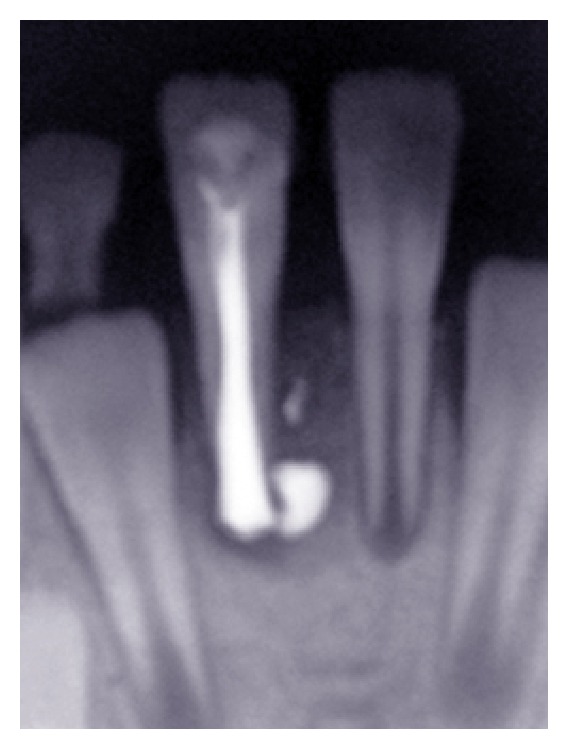
One month follow-up radiograph.

**Figure 6:**
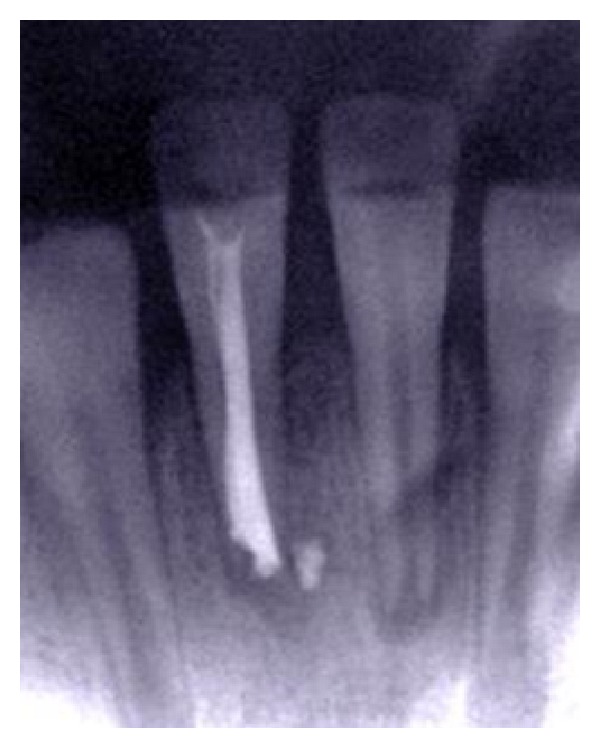
Three-month follow-up radiograph.

Patient was followed up regularly at 1 and 3 months (Figures 5 and 6). Ninety days after the replantation, a noticeable reduction of periapical radiolucency was observed. Thus, in order to reduce the risk of tooth fracture, we decided to fill the canal using an apical plug of calcium enriched mixture (CEM) cement (BioniqueDent, Iran). Therefore, the root canal was washed out with sodium hypochlorite 2.5%, and the CEM cement was placed into the apical 4 mm of the canal. Tooth was obturated using vertical compaction technique with gutta-percha (Gapadent Co., Ltd., Korea) and AH26 sealer (DeTrey, Dentsply, Konstanz, Germany) and then access cavity was restored with composite restoration.

## 3. Clinicoradiographical Follow Up

**Figure 7:**
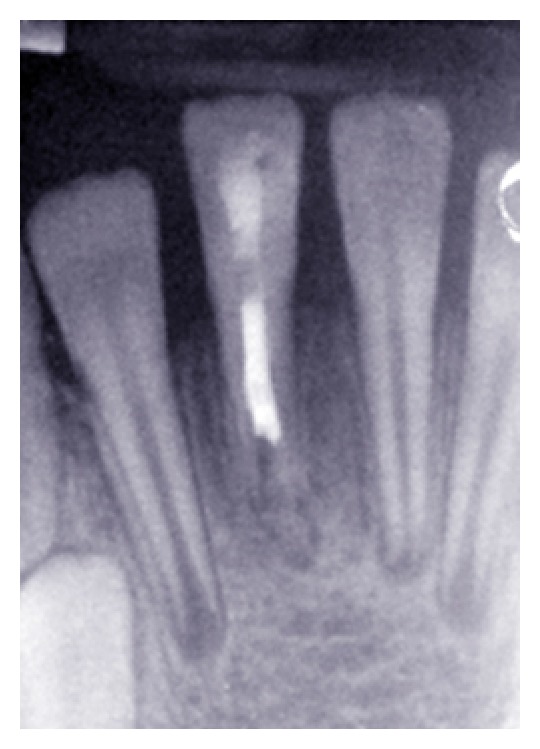
Twelve-month follow-up radiograph; continued root formation is detectable.

**Figure 8:**
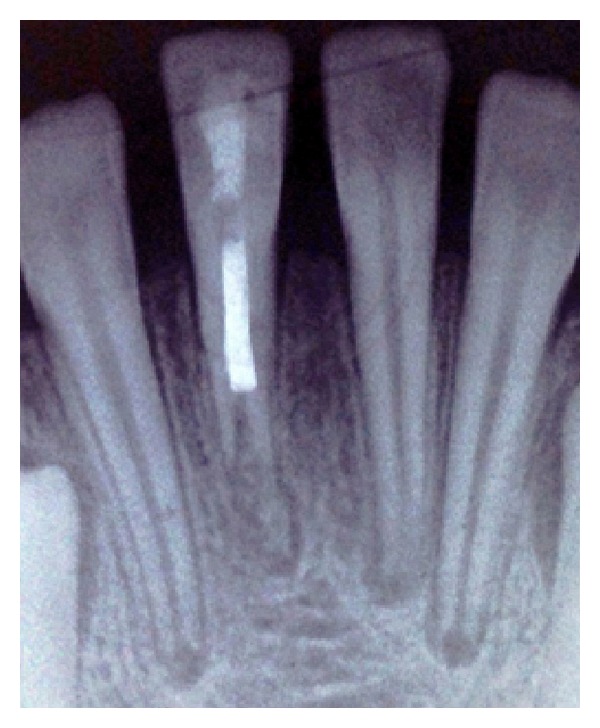
Twenty-month follow-up radiograph; continued root formation is very noticeable.

Tooth mobility and sensitivity to percussion were examined every three months. The percussion tone was evaluated and compared with the adjacent teeth. At twelve-month follow-up radiograph, continued root formation was observed (Figure 7), and at 20 months after replantation the tooth was completely asymptomatic, with physiologic mobility. Also, at that time, continued root formation was very noticeable (Figure 8).

## 4. Discussion

The most important factor that impacts on treatment's outcome of the avulsed teeth is the extraoral dry time [7]. Although immediate replantation is the best available treatment for an avulsed tooth, for a variety of reasons this may not happen [7].

This case report describes a relatively severe, rare traumatic injury to an immature right lower central incisor. According to current literature [3, 12], replanting immature teeth is irrational when the extraoral dry time is more than 60 minutes. They stated that, in such cases, thin dentinal walls of immature teeth, wide dentinal tubules, and the high basal metabolic rate in children lead to an unsuccessful replantation. Although, in our case, the right lower central incisor was replanted after a relatively long extraoral period of time (90 min), the tooth had been placed in a glass of milk.

Before replantation, we soaked the tooth in doxycycline for 5 minutes, because it has been shown that it enhances significantly the pulp revascularization in replanted immature teeth [13, 14].

The prerequisite for healing of an avulsed tooth is absence of infection [7]. Therefore, when increasing periapical radiolucency around the replanted tooth was observed, endodontic treatment to remove infected pulp tissue and stop resorption process was performed.

It has been suggested that placement of an intracanal medicament for a period ranging between 2 weeks and 6 months during root canal therapy of the avulsed teeth could have beneficial effect on the treatment's outcome [7]. In the previous studies several medications such as calcium hydroxide [15], endodontic medication based on steroids and antibiotics (Ledermix paste) [16], andthe mixture of calcium hydroxide and iodoform [17, 18] have been recommended.

Premixed calcium hydroxide paste containing iodoform resorbs from the apical tissues in a short period of time [19]; it is also apparently harmless to permanent tooth germs, does not set to a hard mass, and is easily inserted and removed [19]. Furthermore, it has been demonstrated that placement of calcium hydroxide and iodoform paste (Metapex) for treatment of an avulsed permanent tooth led to continued root-end growth and successful apexification [18].

Therefore, we placed calcium hydroxide and iodoform paste (Metapex) as an intracanal medicament for a relatively long period of time.

Three months after placement of the intracanal medication, periapical radiolucency around the replanted tooth was noticeably reduced. At that time, there were two treatment choices available: (1) long-term placement of calcium hydroxide to create a physiologic hard tissue barrier [20] and (2) creation of a hard tissue barrier with the endodontic biomaterials as the apical plugs [21, 22].

Although the formation of a physiologic hard tissue barrier is quite predictable, it takes anywhere from 3 to 18 months [20], andit has also been shown that long-term calcium hydroxide as a root canal dressing may increase risk of root fracture [23]. Thus, the second option was chosen.

In order to create the apical barrier, CEM cement was used. CEM cement is a biocompatible biomaterial [24] which has been utilized for the apexification and apexogenesis treatments in several studies [25, 26]. It is demonstrated that it has an acceptable sealing ability when it is used to seal the root-end cavities [27] and furcal perforations [28]. Also, it has been shown that, in comparison to mineral trioxide aggregate (MTA), CEM cement's apical plug has superior sealing ability [29].

Torabinejad et al. [30] hypothesized that cementogenic properties of an artificial apical plug might be because of several features such as biocompatibility, alkalinity, and sealing ability. As mentioned before biocompatibility of CEM cement has been demonstrated previously [31]. Also, CEM cement is an alkaline biomaterial (pH > 10.5) [32] with an acceptable sealing ability [27, 33].

One year after replantation of the tooth number 25, continued root formation including an apical segment beyond the artificial apical barrier was observed, and 8 months later the continued root growth was more noticeable. Considering the fact that continued root formation after replantation and root canal treatment in an avulsed tooth has been reported previously [11], it seems that, even in a replanted tooth, a completely disinfected root canal with a tight coronal seal can provide an appropriate condition for activation of hard tissue formation process in periapical tissues. To explain this phenomenon, some possible mechanisms could be considered.

The first mechanism is the role of root organization of Hertwig's sheath; it is possible that this sheath survived and retained its ability to organize root formation when the inflammatory process was eliminated [34, 35].

The second mechanism is the mesenchymal stem cells in the apical papilla of permanent immature teeth; these cells are the source of odontoblasts and can develop root dentin [36]. It has been demonstrated that even if infection of root canal space reaches the periradicular tissues, these stem cells may survive and contribute to tissue regeneration [36].

In the present case, 20 months after replantation, the right lower central incisor was completely asymptomatic and functional; although the last periapical radiograph might make the observer suspicious of replacement resorption, the percussion tone was the same as that of the healthy adjacent tooth, and it was mobile within normal limits.

It is clear that long-term follow up for this tooth is favorable. Also, further studies are needed to evaluate the accurate development mechanism in cases that are like this kind of condition.

## 5. Conclusion

Creation of an appropriate apical barrier following the disinfection of root canal system promoted continued root-end growth in a replanted immature permanent tooth.
